# Next generation sequencing driven successful combined treatment with laparoscopic surgery and immunotherapy for relapsed stage IVB cervical and synchronous stage IV lung cancer

**DOI:** 10.18632/oncotarget.26769

**Published:** 2019-03-12

**Authors:** Clelia Madeddu, Paraskevas Kotsonis, Fabrizio Lavra, Giacomo Chiappe, Luca Melis, Ester Mura, Mario Scartozzi, Antonio Macciò

**Affiliations:** ^1^ Department of Medical Sciences and Public Health, University of Cagliari, Cagliari, Italy; ^2^ Department of Gynecologic Oncology, Azienda Ospedaliera Brotzu, Cagliari, Italy; ^3^ Department of Nuclear Medicine, Azienda Ospedaliera Brotzu, Cagliari, Italy; ^4^ Department of Pathology, Azienda Ospedaliera Brotzu, Cagliari, Italy

**Keywords:** laparoscopic total pelvic exenteratio, immunotherapy, synchronous cancer, cervical cancer, next-generation sequencing

## Abstract

Background: The treatment of patients with multiple synchronous tumors is challenging and complex. The use of next generation sequencing (NGS) may help in identification of germline mutations in genes involved in a common etiology for both tumors thus allowing a common effective therapeutic strategy. Patients and Methods: We describe the unexpected positive results obtained in a young woman with relapsed chemo-resistant stage IVB cervical and synchronous stage IV lung cancer, who underwent an interdisciplinary approach including palliative surgery with laparoscopic total pelvic exenteratio followed by a chemo-immunotherapy protocol with the anti-Programmed Death (PD)-1 antibody nivolumab plus metronomic cyclophosphamide. The treatment choice was based on tumor PD-Ligand 1 assessment and NGS analysis for the identification of potential treatment targets. Outcomes included tumor objective response and patient-centered outcomes (pain, performance status and overall quality of life). Results: Laparoscopic surgery obtained an immediate symptom control and allowed the early start of medical treatment. One month after combined therapy start the patient achieved a significant improvement in performance status, pain, overall Quality of life and after 3 months she resumed working. After 3 and 6 months of treatment we observed an objective dimensional and metabolic response. Currently, after 24 months (and 48 cycles of nivolumab) the patient is continuing to benefit from treatment: she is in complete remission, with good performance status and she is working and leading a self-dependent life. Conclusion: Our study strongly affirms the efficacy of an interdisciplinary approach including surgical and innovative medical strategies based on immunotherapy in patients with advanced chemo-resistant synchronous cervical and lung cancer. The present findings support the use of NGS to drive a targeted rational treatment especially in heavily pre-treated patients.

## INTRODUCTION

Multiple primary tumors that develop in different organs are called synchronous when they are diagnosed within a period of <6 months (IARC definition) or 2 months (SEER database) [1]. Synchronous cancers of the cervix and lung are rarely reported [2]; however, a significantly higher incidence of a secondary lung malignancy has been reported in patients with cervical cancer [3, 4], suggesting an abnormal genetic background and/or a common etiology for both tumors. Some studies have suggested that both cervical and lung cancers may be related to the socioeconomic status and some lifestyle-related risk factors such as smoking. The therapy of patients with multiple synchronous tumors is demanding and frequently a dilemma [1]. The main difficulty is to establish a therapeutic approach that deals with both cancers without increasing toxicity or pharmacological interactions and has no detrimental effects on the final clinical outcome. While surgery or radiation/chemoradiation therapy can cover both malignancies in the case of localized disease, it is often difficult to choose an optimal regimen in advanced disease. Treatment strategies may be based on the prognosis of both tumors, and today on their molecular profile and genetic background, availability of an active systemic regimen for both cancers, and the choice between a radical or palliative approach.

Then, the identification by the modern next generation sequencing (NGS) analysis of germline mutations in genes involved in a common etiology for both tumors may facilitate a common effective therapeutic strategy in patients with synchronous cancers. Moreover, the use of NGS may be fundamental to identify predictive biomarkers of response/resistance to systemic chemotherapy and immune checkpoint inhibitors, as a prelude to the development of a rational treatment strategy, especially in pre-treated patients [5]. In particular, effective treatment in advanced cervical cancer after first-line chemotherapy is not established. In the last years the use of immunotherapy with anti-programmed death (PD)-1 antibody demonstrated to be effective and has been approved for the treatment of metastatic lung cancer [6], while only preliminary promising data in patients with cervical cancer are available [7]. The identification of predictive markers for immunotherapy is evolving. In fact, the programmed death-ligand 1 (PD-L1) expression in tumor sample is recognized as marker for eligibility to immunotherapy in non-small-cell lung cancer (NSCLC) although not univocally [8]. Other gene mutations, especially those involved in mismatch repair (MMR) may predict efficacy of immunotherapy [9]. Here we describe the case of a patient with advanced synchronous cancer of the cervix and lung where the perspective use of the NGS analysis supported the choice of an immunotherapy-based treatment with unexpected positive results.

## RESULTS

**Figure 1:**
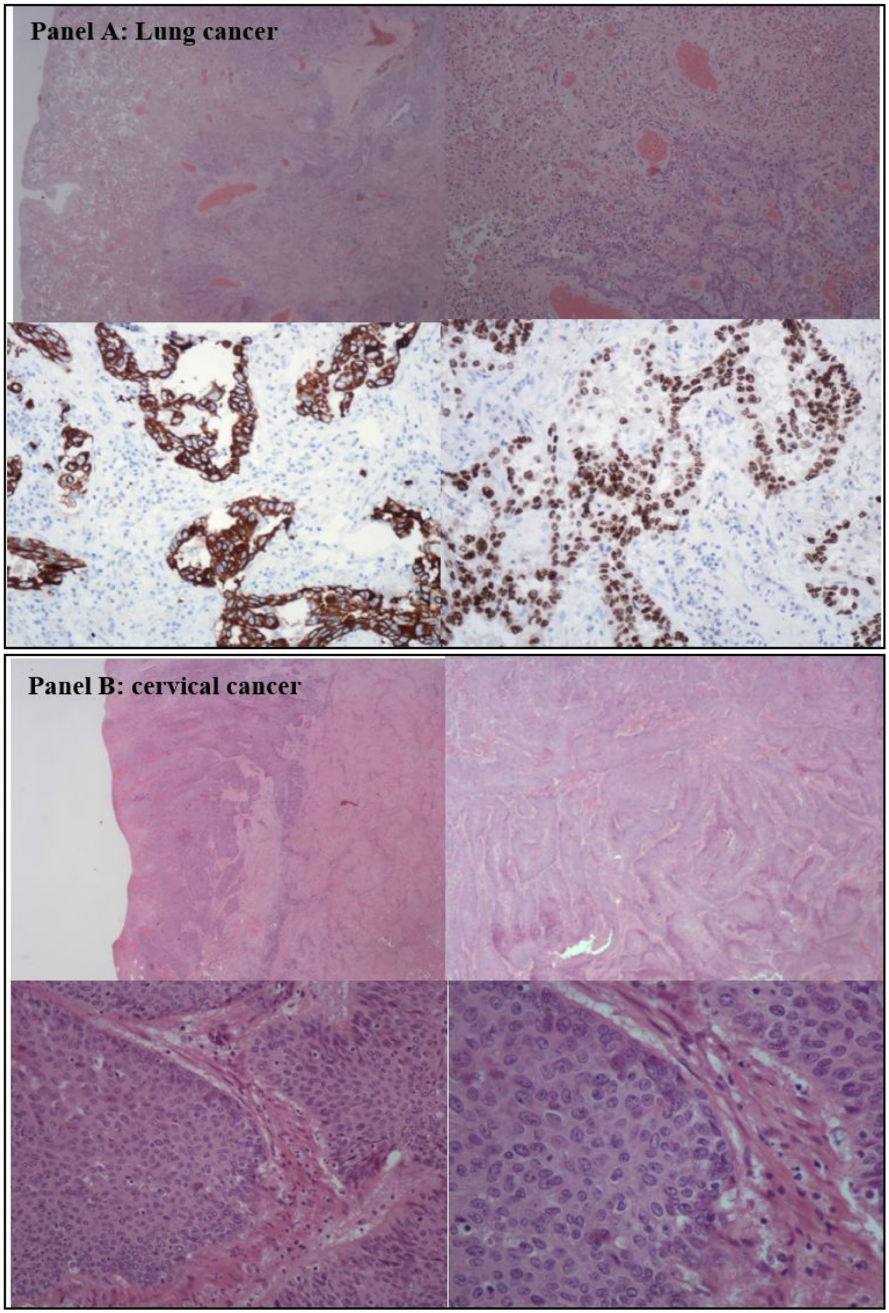
Histological analysis of the lung and cervical cancer (**A**) shows histology of non-small-cell-lung cancer adenocarcinoma. (**B**) shows histological findings of the squamous cervical cancer.

**Figure 2:**
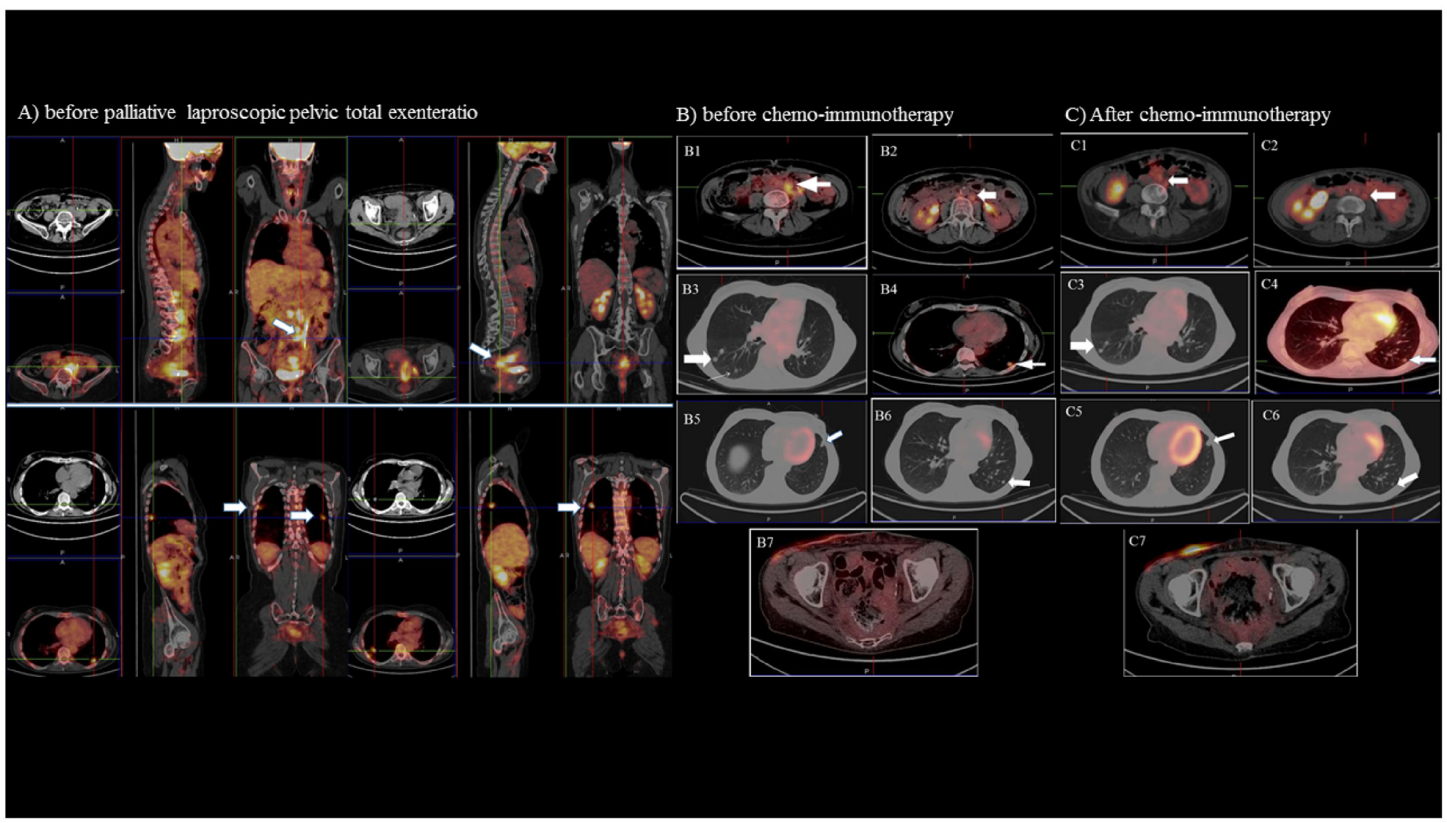
Positron emission tomography (PET) and computed tomography (CT) imaging The imaging of the disease extent before laparoscopic total pelvic exenteratio (Panel **A**) show in particular in the upper scans a large area of intense hypercaptation of the metabolic tracer in the pelvis and in the lombo-aortic and common iliac lymph node (white arrows); in the lower scans multiple positive areas in the lung (apical segment of the right superior lobe, superior segment of the inferior lobe bilaterally, a medium postero-basal segment of the left inferior lobe (white arrows). (Panel **B** and **C**) shows the PET/CT and CT imaging before (panel B) and after (panel C) chemo-immunotherapy of the: 1-2) left lombo-aortic lymphnodes; 3) right lung nodules; 4-6) left lung nodules; 7) pelvic region.

We studied a 48 year-old woman, with a positive history of heavy smoking (100 pack/year), with synchronous advanced cervical cancer (stage IVA) and metastatic NSCLC. The patient oncological history started in February 2016 when at another Unit of Obstetrics and Gynecology, a locally advanced spinocellular cervical cancer has been diagnosed. At that time the magnetic resonance imaging (MRI) of the pelvis detected a neoplasm that measured 42 × 57 × 64 mm and extended to the upper third of the vagina and invaded the parametria (cT2B, Nx: Stage IIB). The thorax computed tomography (CT) scans also showed a lesion measuring 17 × 7 mm localized near the parietal pleura at the superior lobe of the left lung and another contralateral lung lesion in the right superior lobe. The positron emission tomography (PET)/CT confirmed these lesions to be hypermetabolic. A CT-guided biopsy of the lung lesion and its histological examination revealed primary lung adenocarcinoma (Figure 1A). A molecular and biomarker analysis for NSCLC indicated the absence of EGFR, ROS, ALK, and BRAF mutations. Since the tumors were judged to be non-operable, she received 6 cycles of cisplatin plus taxol, potentially effective against both tumors. However, though the NSCLC was stabilized, in September 2016 a clear progression of the cervical tumor was seen. Following another cycle of chemo-radiotherapy, cervical cancer progressed further. Hence, in December 2016 the patient was admitted to our Department of Gynecologic Oncology, Businco Hospital, Azienda Ospedaliera Brotzu, Cagliari. At admission the patient was experiencing strong debilitating symptoms such as severe pain [Visual analog scale (VAS) 9] in the pelvis and lower back associated with heavy spontaneous and malodorous vaginal bleeding and discharge. She had abandoned her job and was unable to take care of the most common home and personal hygiene tasks. Her performance status (PS) was 40 by the Karnofsky and 3 by the Eastern Cooperative Oncology Group (ECOG) scales. A physical examination showed an ulcerative cervical cancer that extended to the lower third of the vagina with abundant malodorous leucorrhea and easy bleeding. Laboratory analyses showed anemia (Hb 8.6 g/dl, normal range 12–16 g/dl) and raised indices of inflammation (C-reactive protein, CRP, 34 mg/L (normal range 0–1 mg/L; Interleukin (IL)-6 158 pg/mL, normal range 0–15 pg/ml). An abdominal-pelvic MRI showed a cervical neoplasm measuring 42 × 36 × 32 mm that extended to the left paracervical side embodying the ureter causing hydronephrosis, invaded the posterior wall of the bladder and rectum, and extended to the lower third of the vagina. Also enlarged confluent lymph nodes measuring 61 × 23 mm at the left para-aortic level were observed. The clinical pre-surgical stage was IVa. The 18^F^ PET/CT demonstrated a large area of intense hypercaptation of the metabolic tracer in the pelvis and multiple areas in the lung (apical segment of the right superior lobe, superior segment of the inferior lobe bilaterally, medium postero-basal segment of the left inferior lobe) and lombo-aortic and common iliac lymph nodes (Figure 2A).

The patient required palliative surgery, and we opted for a laparoscopic total pelvic exenteration (LTPE). Compared to an open procedure, LTPE is more effective in decreasing morbidity, hospital stay, and tumor recurrence, and in improving cancer-related survival, quality of life (QL) and possibility of starting early other antineoplastic treatments. We, therefore, performed LTPE with total vaginectomy. The first case of LPTE was performed by Pomel *et al.* [10] in 2003 to treat a relapse in cervical cancer. Since then, the LTPE has been successively performed in selected patients at some experienced laparoscopic centers [11]. In our case the surgery included the excision of the sigma-rectum, bladder, uterus, and total vagina as a single mass, extracted via the vaginal route, followed by extracorporeal reconstruction by cutaneous uretero-ileostomy according to the Bricker procedure and fecal diversion by an end-colostomy (Supplementary Figure 1). The surgery lasted 400 minutes. No intraoperative or perioperative complications occurred. No wound hematoma, infection, or delayed bleeding was observed postoperatively. The patient was discharged from the hospital on postoperative day 5 in good condition. The definitive histological examination (Figure 1B) showed a squamous cell carcinoma of the uterine cervix with wide necrosis and eso-endocervical ulceration, infiltrating the stroma until the serosa, involving the parametria bilaterally, extending to 2/3 of the endometrial cavity, and deeply infiltrating the bladder and rectum musculature without mucosal involvement.

At this point, considering that the patient was heavily pre-treated and showed resistance to standard cytotoxic chemotherapy and radiotherapy, in order to determine genomic predisposing variants for both tumors as well as predictive biomarkers useful to establish a rationale targeted approach, we performed simultaneously both: a) the analysis of PD-L1 expression both in cervical and lung cancer; b) the whole exome NGS of germinal DNA (gDNA) from the patient’s peripheral blood.

Based on the results of PD-L1 assessment and NGS that suggested a potential response to immunotherapy, we started an immunotherapy regimen with the anti PD-1 antibody, nivolumab (3 mg/kg every two weeks). Based on the high levels of IL-6 and CRP, both of which are potentially immunosuppressive, we also opted for a metronomic dose of cyclophosphamide (50 mg/day continuously) as an antiblastic and immunomodulating agent [12]. Patient received also a supportive therapy for specific symptoms control, in detail a combined nutritional anticachectic regimen with L-carnitine, lactoferrin, aminoacids and antioxidants [13] alongside the appropriate analgesic drugs for pain.

### Germline whole exome sequencing analysis

In search of genomic variants mediating predisposition and etiology of synchronous cancer and mutations predictive of response to treatment, we performed NGS of the patient’s germline DNA. The whole exome sequencing identified a total of 11344 genetic variants of which 2388 occurred in non-intronic regions (among them 374 missense, 663 3′UTR, 344 5′UTR, 196 synonymous, 186 splicing, 184 upstream, 143 downstream, 130 non-frameshift, 56 frameshift, 52 noncoding RNA, and 10 stop gain variants).

The analysis was prioritized to identify genetic mutations in genes related to cancer predisposition, genes involved in DNA repair, genes associated with tobacco exposure/nicotine dependence and immune system modulation. In particular, variants reported in the general population with a minor allele frequency (MAF) lower than 1% (MAF<0.01) according to dbSNP database were considered.

In detail, the NGS demonstrated 32 genetic variants in 25 genes related to DNA repair/cancer predisposition: according to dbSNP database we found 16 single nucleotide polymorphisms (SNP) (Supplementary Table 1). In particular, these included a germline single nucleotide variation (SNP: rs61755655) in exon 2 of the MLH3 gene (c.408T > C), related to Lynch syndrome and a single nucleotide variation (missense variants) (SNP: rs140825795) in the exon 1 of the RAD51D gene (c.26G > C; p.Cys9Ser), related to a form of hereditary cancer-predisposing syndrome, according to ClinVar database (https://www.ncbi.nlm.nih.gov/clinvar/) (Table 1). These genes have been also associated with an increased risk of cervical and lung cancer [14–16]. Additional 6 SNPs in 5 genes involved in nicotine dependence were found, of which 3 with a MAF < 0.01 (Table 1 and Supplementary Table 1). These included a single missense nucleotide variation (SNP: rs56344740) in the exon 6 of the gene encoding for the cholinergic receptor nicotinic alpha 2 subunit (CHRNA2) (c.1434C>A) associated with a mutation in the third nucleotide of the 478th triplet codon (Asp478Glu) that lead to an altered protein function [17].

**Table 1:** Single nucleotide polimorphysm (SNP) with known clinical significance

dbSNP	Gene ID	Locus	Reference sequence	Coding DNA sequence	Protein sequence	MAF (dbSNP database)	Genotype	Clinical Significance (ClinVar)	Effect	PMID
rs61755655	MLH3, mutL homolog 3	chr14:75515951	NM_001040108.1	c.408T>C	p.Asp136=	0.0020/10 (1000 Genomes)	HET; synonimous	Conflicting interpretations of pathogenicity; Lynch syndrome; MLH3-Related Lynch Syndrome	Cancer susceptibility	17494052
rs140825795	RAD51D, RAD51 paralog D	chr17:33446607	NM_002878.3	c.26G>C	p.Cys9Ser	0.0004/42 (ExAC)	HET; missense	Conflicting interpretations of pathogenicity: Breast-ovarian cancer, familial 4, Hereditary cancer-predisposing syndrome	Cancer susceptibility Cervical cancer	2182226; 24879636; 24139550
rs138463559	MSH6, mutS homolog 6	Chr2: 48030462	NM_000179.2	c.3173-97T>C		0.0008/4 (1000 Genomes)	HET; Intronic	Likely benign	Colon cancer susceptibility	18523027
rs56344740	CHRNA2, cholinergic receptor nicotinic alpha 2 subunit	Chr8:27320526	NM_000742.3	c.1434C>A	p.Asp478Glu	0.0016/8 (1000 Genomes)	HET; missense	Benign/Likely benign	Nicotine dependence/metabolism	24950454

Results are reported following international guidelines (ref 9). SNPs were matched with dbSNP as population database (http://www.ncbi.nlm.nih.gov/snp) and with ClinVar as disease database (http://www.ncbi.nlm.nih.gov/clinvar). Abbreviations: Chr, Chromosome; HET, heterozygosis.

Moreover, 13 SNPs (MAF<0.01 for all) in genes reported to be involved in the immune response modulation were found (Supplementary Table 1). In particular, we observed a splicing variants (rs372373486) in the intronic region of the gene encoding for DEAD-box-helicase 41 (DDX41), a protein involved in the innate immune response [18]; an upstream single nucleotide variation (rs139470452) in the gene encoding for the sialic acid binding Ig like lectin 1, a type I transmembrane protein expressed by macrophages and involved in mediating cell-cell interactions [19]; a stopgain single nucleotide variation (rs148281714) in the gene encoding the leukocyte receptor tyrosine kinase, which belongs to the ros/insulin receptor family members expressed in B lymphocyte progenitors [20]; a splicing variants (rs779116887) in the gene encoding for the hepatitis A virus cellular receptor 2, a Th1-related protein involved in the regulation of macrophage activation, inhibition of Th1-mediated immune responses, and promotion of immune tolerance [21]; a non-frameshift deletion variants (rs773770060) in the exon 5 of the gene encoding for the IL-27, a protein that drive expansion and interferon gamma production by naïve CD4 T cells [22]; a non-frameshift deletion variation (rs774578296) of the gene encoding for the IL-4 receptor that binds interleukin 4 to promote differentiation of Th2 cells [23]; a 3UTR deletion variation (rs559799140) in the gene encoding for the nuclear factor, erythroid 2 like 2, which is involved in the regulation of the myeloid suppressive cells and have been correlated with PDL1 expression in lung cancer [24].

### PDL1 assessment

The immunohistochemical analysis revealed PDL1 expression >50% in the lung (immunoreaction intensity 3+) and about 30% in the cervical tumors (immunoreaction intensity 2+).

### Patient response to immunotherapy-based combination regimen

Five days after surgery, the patient was discharged with improved general conditions (PS 2; pain VAS 6). Fifteen days after surgery, based on the results of PDL1 assessment and NGS that suggested a potential response to immunotherapy, we started a combination regimen with the anti-PD1 antibody nivolumab 3 mg/kg every two weeks in combination with metronomic chemotherapy (cyclophosphamide 50 mg/day continuously). One month after treatment start the patient achieved a significant improvement in PS (ECOG 1), pain (VAS 0), overall QL, and Hb levels associated with a decrease in CRP and IL-6 levels. The treatment was well tolerated, and no adverse effects of any grade were reported by the patient. After 3 months, the patient resumed working and taking care of herself. Evaluation of the tumor objective response by CT and PET/CT after 3 and 6 months of combined treatment, demonstrated a dimensional and metabolic response at the level of the pelvis and the multiple metastatic lung lesions. Further PET/CT scans after 9 and 12 months of treatment showed a complete response with no evidence of lesions with high metabolic activity (Figure 2B–2C). The MRI of the pelvis after 12 months of treatment confirmed the absence of a neoplastic residual/relapse. Currently, after 48 cycles of nivolumab, the patient is continuing to benefit from treatment: she is in complete remission, has normal blood results (Hb 13.5 g/dl), and normal levels of chronic inflammatory markers. Her PS is 1, and she is working and leading a self-dependent life (Supplementary Figure 1). The last CT as well as PET/CT scans confirmed the objective and metabolic response.

## DISCUSSION

The present report describes a unique case in literature of a patient with relapsed synchronous cervical and lung cancer who underwent a rationale systemic treatment, including the most modern antineoplastic approach, based on the use of perspective NGS with an unexpected positive response both in terms of survival and QL.

Indeed, in literature the description of exceptional responders, i.e. patients who achieve an extraordinary response to treatments, “are emerging as frontrunner cases for NGS analysis” and represent a relevant source for the discovery of novel genomic alterations predictive for specific tailored approaches [25].

Notably, in our case the patient was firstly treated with optimal palliative laparoscopic surgery; thus, we affirm how LTPE when performed in selected patients with cervical cancer can have a significant role in opening the road to other life-prolonging antineoplastic treatments [26], allowing also their early start [27]. Thus, tumor exenteration which was once considered only for the purpose of palliation without increasing the life expectancy is not only capable of improving the patient general well-being but can give also the time to allows the use of further innovative therapeutic strategies. Moreover, even if severe symptoms able to interfere with the physical, psychological, emotional well-being of the patient were sufficient to decide for a palliative surgery, the presence of concomitant synchronous neoplasms made such surgical approach mandatory. In fact, in this case palliation of one tumor’s symptoms could help in the treatment of the other tumor, which can potentially benefit from effective and specific systemic drugs. Therefore, although symptoms relief, reported in 67–90% of patients [28], and not just survival, is the best measure of success after palliative exenteration, a substantial improvement in the QL associated with life prolongation cannot be excluded when other interdisciplinary treatments are introduced. Here, we confirm the success of this interdisciplinary approach, which depends on the shared skills of the operating teams and the harmony between them.

Consistently, in our case immediately after having obtained a significant improvement of the patient general well-being with surgery, we performed a perspective NGS analysis of the germinal whole exome in order to find predisposing genetic factors for the synchronous cancers and even more genetic variants of predictive parameters useful to choose the best and most effective therapeutic strategy. Indeed, since the patient was heavily pre-treated and progressed under standard cytotoxic agents both for cervical and lung cancer, the choice of an effective treatment was challenging and complex. Then, considering that anti-PD1 antibodies are approved for the treatment of NSCLC [6] and showed preliminary positive results in cervical cancer [7], we searched with NGS genetic variants that may be predictive of immunotherapy efficacy. Additionally, being already known that in NSCLC the tumor expression of PD-L1 represents a parameter for eligibility to such drugs, we have assessed it also in the cervical tumor sample and we found a hyperexpression in both cancers (>50% in NSCLC sample and about 30% in cervical cancer).

NGS analysis revealed both the presence of some significant gene variants known to predispose to lung and cervical cancers as well as to nicotine dependence and chronic lung diseases thus hypothesizing a common etiology and then a possible common therapeutic strategy, and notably also demonstrated genetic variants predictive of response to immunotherapy, thus supporting strongly our treatment choice. In particular, we identified some germline mutations in several genes involved in mismatch repair (MMR), which have been associated with increased efficacy of immunotherapy [9]. Mutations in MMR genes impair DNA repair, which can lead to microsatellite instability (MSI). MSI, which is believed also to be a predictor of chemoresistance [9] as we observed in our patient, is associated with high frequencies of non-synonymous and frameshift mutations that result in increased neo-antigen formation. Neoantigens are recognized as foreign by the immune system and induce a stronger, longer, and more specific immune response against the tumor. Some authors have shown that mutations in DNA repair and replication genes are associated with a higher mutational load and correlated with increased response to the PD-1 blockade [29–31]. Based on this evidence, very recently the FDA recognized MSI as a predictor of sensitivity for immunotherapy approaches. Additionally, the genomic analysis in our case also identified significant SNPs in DEAD-box-helicase 41, a protein involved in the innate immune response, which is related to Interferon signaling [32], and other genes for cytokines, chemokine receptors, and other factors, such as the the sialic acid binding Ig like lectin 1, the hepatitis A virus cellular receptor 2 and the nuclear factor erythroid 2 like 2, that are involved in macrophage activation whose roles in modulating the response to immunotherapy has already been hypothesized in literature [33–35].

On the basis of all the above findings, we provide a strong molecular and genetic rationale for the choice of a treatment protocol which consisted of immunotherapy with antiPD1 antibody also for our patient affected by chemoresistant advanced cervix cancer and synchronous NSCLC, after palliative laparoscopic surgery for the cervical cancer relapse. We combined the immune checkpoint inhibitor nivolumab with metronomic cyclophosphamide. Such choice was based on the evidence that our patient presented a cancer-related severe chronic inflammation, which is able to impair the specific immune response mediated by T cell [12]. In fact, metronomic chemotherapy and in particular cyclophosphamide showed anti-inflammatory and immunomodulating actions that determine a synergism with immunotherapy [12]. In particular, cyclophosphamide is capable to modulate the tumor microenvironment by inhibiting the immunosuppressive macrophagic activity and the synthesis of proinflammatory mediators (prostaglandin and cytokines) [36]. As result of our therapeutic approach, and after 48 administrations of nivolumab, the patient has a complete objective and metabolic response, and she is surviving with a good PS and a marked improvement in QL, for more than 24 months after the LTPE was performed. At this regard it should also be noted that our patient passed the median duration of response observed in the largest trials on the treatment with anti PD1 antibody in patients with NSCLC adenocarcinoma [6] and, more significantly, the median duration of progression-free survival reported cervical cancer patients treated with pembrolizumab in one clinical trial [7] and few case series [37, 38].

In conclusion, the present case strongly affirms the need for an interdisciplinary approach by combining surgical and medical strategies and supports the use of NGS to drive a targeted rational treatment in heavily pre-treated patients with cervical cancer synchronous to lung cancer.

Future perspective larger research with implementation of both germinal and somatic genomic sequencing are warranted to expand the development of new targeted immunotherapy-based approaches, as those described in the present paper.

## MATERIALS AND METHODS

### Oversight

The study protocol was approved by the local institutional review board and conducted according to the principles expressed in the Declaration of Helsinki. The patient provided written informed consent for the treatment protocol and also for genomic analysis as well as for the case report and accompanying images publication.

### Germline whole exome sequencing analysis

Whole exome NGS of the germinal DNA obtained from patient’s peripheral blood cells was performed using the Illumina HiSeq NGS platform that can detect mutations (single nucleotide polymorphism) and copy-number variations. We focused on genes related to cancer predisposition, DNA repair, tobacco exposure /nicotine dependence as well as on genetic variants of parameters that could be predictive of response to targeted/immunological strategies potentially useful in our patient who showed to be resistant to conventional chemotherapy. We focused on rare variants, with MAF < 0.01, which can exert a central role in the generation of complex traits and be responsible of heritability not explained by common variants. Results of NGS are reported following international guidelines [39].

### PDL1 assessment

We assessed the expression of PDL1 in specimens of primary lung and cervical cancers, by immunohistochemistry.

### Surgical technique

Details of the procedure are reported in the Supplementary Appendix.

### Clinical, laboratory and quality of life outcomes

The patient’s tumor objective response was monitored as per the iRECIST criteria [40]. It was evaluated every 4–6 months by pelvic MRI, CT and PET/CT. Toxicity was evaluated by the National Cancer Institute Common Terminology Criteria for Adverse Events, version 4.03. In addition to the routine laboratory analyses, we also evaluated changes in circulating levels of the proinflammatory cytokine IL-6 (by enzyme-linked immune assay) and CRP. We also determined PS, pain (VAS) and QL by the European Organization for Research and Treatment of Cancer quality of life Questionnaire (EORTC-QLQ)-C30. All assessments were made every month.

## SUPPLEMENTARY MATERIALS FIGURE



## Abbreviations

NGS: next generation sequencing
PD: programmed death
PD-L1: programmed death-ligand 1
NSCLC: non-small-cell lung cancer
MMR: mismatch repair
MRI: magnetic resonance imaging
CT: computed tomography
PET: positron emission tomography
VAS: visual analogue scale
PS: performance status
ECOG: Eastern Cooperative Oncology Group
CRP: C-reactive protein
LTPE: laparoscopic total pelvic exenteration
QL: quality of life
gDNA: germinal DNA
(MAF): minor allele frequency
IL: Interleukin
SNP: single nucleotide polymorphisms
CHRNA2: cholinergic receptor nicotinic alpha 2 subunit
DDX41: DEAD-box-helicase 41
MSI: microsatellite instability
EORTC-QLQ: European Organization for Research and Treatment of Cancer quality of life Questionnaire
