# Supplementary material for: Next generation sequencing driven successful combined treatment with laparoscopic surgery and immunotherapy for relapsed stage IVB cervical and synchronous stage IV lung cancer
Source: Oncotarget. 2019 Mar 12;10(21):2012–21. doi: 10.18632/oncotarget.26769 (PMC6459345; doi:10.18632/oncotarget.26769)
Supplement: Supplementary file 1 [file oncotarget-10-2012-s001.pdf]

Next generation sequencing driven successful combined treatment with laparoscopic surgery and immunotherapy for relapsed stage IVB cervical and synchronous stage IV lung cancer

SUPPLEMENTARY MATERIALS

Surgical technique

After anesthesia, broad-spectrum antibiotics were administered. We started the surgery with a diagnostic laparoscopy to confirm the unresectability of the tumor by assessing how it was attached to the pelvic wall and the adjoining vessels. A 12-mm port was placed in the umbilicus, and a pneumoperitoneum not higher than 14 mmHg was established and maintained throughout the surgery. Intra-abdominal visualization was achieved using a 10 mm, 0 and 30° telescope varying according to the stages of the surgery (Karl Storz, Tuttlingen, Germany). The technique used involved placing three additional trocars: a 12-mm trocar both in the right lateral side and in the left lateral side, and a 5-mm trocar in the sovrapubic area. We started with coagulation and cut the round ligaments using LigaSureTM (Covidien, Minneapolis, Minnesota, USA). The cut was extended anteriorly, staying medial to the obliterated umbilical vessels, to allow entry into the retroperitoneal space. We continued posteriorly until the infundibulopelvic ligament was coagulated and sectioned. With the cleavage plans in place, we ascertained the possibility of carrying out the intervention. The ureter was visualized and keeping it laterally; the pararectal space was dissected. Hemostatic clips were positioned bilaterally in the internal iliac artery at the origins of the inferior gluteal arteries. The uterosacral and cardinal ligaments were coagulated and cut at their origins. Then, following the avascular plane in front of the Toldt's fascia and posterior to the mesocolon, the sigmoid mesentery was opened to access the presacral space posterior to the rectum. The base of the mesocolon was separated from the immediately distal mid colic vessels to the sacral promontory while ensuring continuity of the marginal arcade. The sigmoid colon was then dissected off the lateral pelvic wall, and the inferior mesenteric vessels were clipped and cut. Proximally the colon was stapled with a linear stapler and cut, in the same manner as the rectum distal to the tumor. After entering the pre-sacral space, a complete mesorectal excision was performed. The dissection posterior to the rectum continued till the levator ani was reached. The laparoscopic approach allowed an optimal view at this stage of the surgery. A portion of the rectum was then isolated and prepared. The rectum, including an oncologically appropriate distal margin, was resected near the anus using a laparoscopic stapling system. At this stage using a Harmonic Ace (Ethicon Endo-Surgery, Inc., Cincinnati, OH), the urachus was cut off. The dissection was continued in the paravesical plane to reach the levator ani muscle; the bladder was dissected off the anterior abdominal wall to enter the cave of Retzius. The tissues around the bladder and ureter were dissected, cutting the lateral bladder ligaments and thereby exposing the two organs. The dissection was not stopped until reaching the level of the levator ani muscle. At this stage, the urethra and the ureter were cut. An adequate margin of the vagina was ensured before performing a very low colpotomy using monopolar scissors. The uterus, adnexa, bladder, and rectum in a unique block were extracted through the vagina as well as the resected segment of the sigma-rectum. The vaginal cuff was then closed with an external suture using a continuous absorbable suture. A bilateral pelvic and lombo-aortic lymphadenectomy was then performed up to the inferior mesenteric artery. No vascular or visceral injury and no intraoperative bleeding occurred during the surgery. Additionally, no complications were noted. The estimated blood loss was 100 mL. The pelvis was irrigated with an aqueous tumoricidal betadine solution. Then, a 4-cm vertical umbilical incision was made, and a cutaneous ureterostomy was performed according to the Bricker's procedure on the right side of the low abdominal wall. An end colostomy was also performed on the left side. The operation time was 400 min. No wound hematoma, infection, or delayed bleeding was observed postoperatively. The patient was discharged from the hospital on postoperative day 5 in good condition.

Supplementary Table 1: SNP of gene associated with DNA repair, nicotine metabolism and immune response with uncertain significance. See Supplementary_Table_1

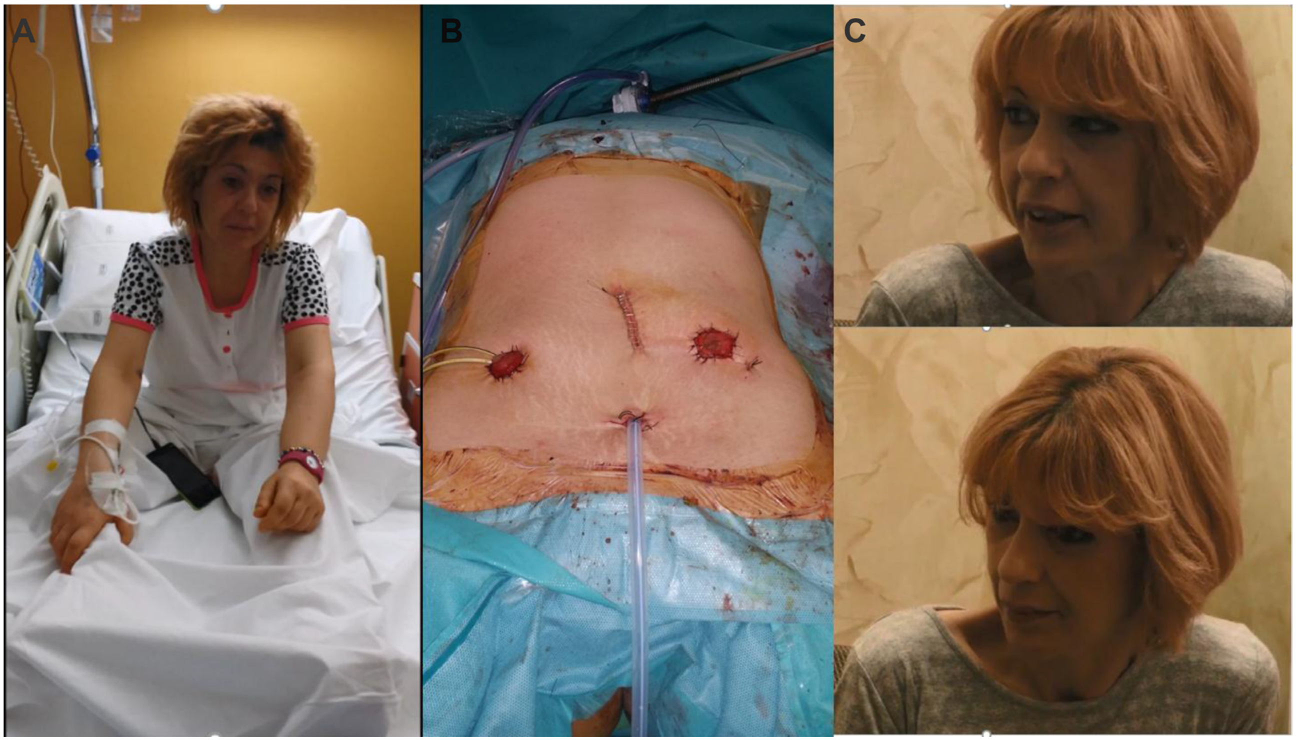

Supplementary Figure 1: Impact of the interdisciplinary approach on the patient general status and body image. (**A**) Patient general status before palliative laparoscopic pelvic total exenteratio, (**B**) imaging of the abdomen after surgery showing the end-colostomy (left) and cutaneous ureterostomy (right) location and (**C**) patient general status after 36 cycles of chemo-immunotherapy regimen.
